# Direct comparison of serial B-type natriuretic peptide and NT-proBNP levels for prediction of short- and long-term outcome in acute decompensated heart failure

**DOI:** 10.1186/cc9398

**Published:** 2011-01-05

**Authors:** Markus Noveanu, Tobias Breidthardt, Mihael Potocki, Tobias Reichlin, Raphael Twerenbold, Heiko Uthoff, Thenral Socrates, Nisha Arenja, Miriam Reiter, Julia Meissner, Corinna Heinisch, Sybille Stalder, Christian Mueller

**Affiliations:** 1 Department of Internal Medicine, University Hospital Basel, Petersgraben 4, 4053 Basel, Switzerland; 2 Department of Cardiology, University Hospital Basel, Petersgraben 4, 4053 Basel, Switzerland

## Abstract

**Introduction:**

Monitoring treatment efficacy and assessing outcome by serial measurements of natriuretic peptides in acute decompensated heart failure (ADHF) patients may help to improve outcome.

**Methods:**

This was a prospective multi-center study of 171 consecutive patients (mean age 80 73-85 years) presenting to the emergency department with ADHF. Measurement of BNP and NT-proBNP was performed at presentation, 24 hours, 48 hours and at discharge. The primary endpoint was one-year all-cause mortality; secondary endpoints were 30-days all-cause mortality and one-year heart failure (HF) readmission.

**Results:**

During one-year follow-up, a total of 60 (35%) patients died. BNP and NT-proBNP levels were higher in non-survivors at all time points (all *P *< 0.001). In survivors, treatment reduced BNP and NT-proBNP levels by more than 50% (*P *< 0.001), while in non-survivors treatment did not lower BNP and NT-proBNP levels. The area under the ROC curve (AUC) for the prediction of one-year mortality increased during the course of hospitalization for BNP (AUC presentation: 0.67; AUC 24 h: 0.77; AUC 48 h: 0.78; AUC discharge: 0.78) and NT-proBNP (AUC presentation: 0.67; AUC 24 h: 0.73; AUC 48 h: 0.75; AUC discharge: 0.77). In multivariate analysis, BNP at 24 h (1.02 [1.01-1.04], *P* = 0.003), 48 h (1.04 [1.02-1.06], *P *< 0.001) and discharge (1.02 [1.01-1.03], *P *< 0.001) independently predicted one-year mortality, while only pre-discharge NT-proBNP was predictive (1.07 [1.01-1.13], *P *= 0.016). Comparable results could be obtained for the secondary endpoint 30-days mortality but not for one-year HF readmissions.

**Conclusions:**

BNP and NT-proBNP reliably predict one-year mortality in patients with ADHF. Prognostic accuracy of both biomarker increases during the course of hospitalization. In survivors BNP levels decline more rapidly than NT-proBNP levels and thus seem to allow earlier assessment of treatment efficacy. Ability to predict one-year HF readmission was poor for BNP and NT-proBNP.

**Trial registration:**

ClinicalTrials.gov identifier: NCT00514384.

## Introduction

Acute decompensated heart failure (ADHF) is the leading cause of hospitalization in adults over 65 years [1]. Despite medical progress, ADHF is still the most costly cardiovascular disorder in Western countries and is associated with a very poor prognosis [1-3].

Early prediction of a patient's clinical course is pivotal for selecting appropriate management strategies for patients with ADHF. However, risk stratification in these patients is still difficult. The tools used for the evaluation of disease severity and prognosis in the past have been criticized because epidemiological and clinical factors like age, New York Heart Association (NYHA) functional class, or Killip class were shown to be inadequately sensitive [4]. Left ventricular ejection fraction (LVEF) determined by echocardiography was once considered a reliable surrogate prognostic marker [5]. Recent reports, however, have demonstrated that about 50% of patients admitted with ADHF have a preserved LVEF [6].

B-type natriuretic peptide (BNP) and N-terminal pro B-type natriuretic peptide (NT-proBNP) are quantitative markers of cardiac wall stress [7,8]. Both natriuretic peptides (NPs) have been shown to accurately mirror heart failure (HF) severity and to correlate well with NYHA classification [9,10]. BNP and NT-proBNP are cleaved in equimolar amounts from proBNP; thus, NP levels correlate with each other [11]. Despite the considerable similarities between the two NPs, their different half-lives and different modes of degradation argue for a separate analysis and make a direct comparison indispensable.

In patients with HF, serial evaluations of BNP and NT-proBNP levels may be useful for guiding therapy decisions by indicating the need for treatment intensification [12-18]. It is, however, unknown whether BNP and NT-proBNP differ in their utility to risk-stratify patients with ADHF. Also, little is known regarding the earliest time point for reliable assessment of treatment efficacy and prognosis. Therefore, the objectives of this study were (a) to define BNP and NT-proBNP plasma concentration profiles from admission to discharge in order to establish the more appropriate timing for these measurements, (b) to assess the role of BNP and NT-proBNP sequential measurement as a marker of clinical improvement of patients with ADHF in response to therapy, and (c) to compare the prognostic utility of BNP and NT-proBNP in this setting.

## Materials and methods

### Setting and study population

One hundred seventy-one patients who presented with ADHF at the emergency departments (EDs) of the University Hospital Basel, Cantonal Hospital Lucerne, and Cantonal Hospital Aarau (all in Switzerland) between August 2007 and September 2008 were enrolled in this study.

During the first hours of hospital presentation, the diagnosis of ADHF was established by the ED resident and ED assistant medical director in charge. In several cases, a board-certified cardiologist was consulted for a confirmation of the diagnosis and for an echocardiography study. To be eligible for study inclusion, patients had to present with ADHF expressed by acute dyspnea NYHA class III or IV and a BNP level of at least 500 pg/mL. The diagnosis of ADHF was additionally based on typical symptoms and clinical findings supported by appropriate investigations such as electrocardiogram, chest x-ray, and Doppler echocardiography as recommended by current guidelines of the American College of Cardiology/American Heart Association and the European Society of Cardiology [19,20]. The study team had no influence on diagnosis or medical treatment.

Patients who required immediate admission to the intensive care unit (ICU) were excluded because of the extensive differences in patient characteristics, disease severity, co-morbidity, and options for treatment monitoring and therapies applied between ICU and ED patients [21]. Acute coronary syndrome was also an exclusion criterion. One year after study inclusion, patients (or, in case of death, their relatives or general practitioner) were contacted by telephone and outcome data were ascertained. The primary endpoint was 1-year all-cause mortality. The secondary endpoint was 1-year HF hospitalization. The study was carried out in accordance with the principles of the Declaration of Helsinki and was approved by the Ethics Committee of Basle (EKBB). Written informed consent was obtained from every patient.

### Biochemical measurements

Blood samples were obtained at presentation to the ED, at 24 hours, at 48 hours, and prior to hospital discharge (mostly during the last day of hospitalization). Treating physicians had access to initial (ED) NP levels but were blinded to serial NP levels. Blood samples were collected in plastic tubes containing, ethylenediaminetetraacetate placed on ice, and centrifuged at 3,000 rpm.

BNP concentrations were determined with the AxSYM BNP assay (Abbott Laboratories, Baar/Zug, Switzerland) [22]. The coefficients of variation within an assay are 6.0%, 4.3%, and 5.1% for concentrations of 108, 524, and 2,117 pg/mL, respectively, and the respective coefficients of variation between assays are 8.1%, 7.5%, and 10%.

Plasma levels of NT-proBNP were determined with the Elecsys proBNP assay (Roche Diagnostics, Basel, Switzerland) [23]. The intra-assay coefficients of variation are 2.4% and 1.8% at 355 and 4,962 pg/mL, respectively, and the respective interassay coefficients of variation are 2.9% and 2.3%.

Cardiac troponin T (cTn) measurement was performed with the use of the Elecsys 2010 system (fourth generation; Roche Diagnostics) with a limit of detection of 0.01 μg/L, a 99th percentile cutoff point of less than 0.01 μg/L, and a coefficient of variation of less than 10% at 0.035 μg/L.

Determination of creatinine levels was carried out with a Hitachi 917 system (Boehringer Ingelheim, Ingelheim, Germany) and Wako Creatinine F L-Type, Stable Liquid-Type reagent F DAOS (Wako Chemicals GmbH, Neuss, Germany) reagents. The measurable range of this enzymatic assay is 0.05 to 100 mg/dL, the normal ranges are 0.55 to 1.10 mg/dL (49 to 97 μmol/L) for men and 0.47 to 0.90 mg/dL (40 to 80 μmol/L) for women, the coefficient of variation is 5%, and the accuracy is ± 10%.

Determination of aspartate aminotransferase (ASAT) was performed with a Hitachi 917 system and with Cobas reagents from Roche Diagnostics (Mannheim, Germany). The measuring range of this assay is 4 to 800 U/L, the analytical sensitivity (lower detection limit) is 4 U/L, the coefficient of variation within an assay is 1.8% at 58 U/L, and the coefficient of variation between assays is 3.2% at 58 U/L.

### Points in time of natriuretic peptide determination

Several studies addressing serial measurements of NPs in patients with ADHF [24-26] described a first notable decrease in NP levels at 24 hours, followed by a nadir at 48 hours and a stable phase during the remaining hospitalization. These observations clearly demonstrated that the major decrease in NP levels in ADHF patients responding to HF therapy occurs during the first 48 hours of hospitalization and appropriate medical treatment. Concomitantly, the best prognostic information by NP measurements in ADHF was obtained prior to hospital discharge [27,28]. Thus, the choice of our sampling points in time was done in order to compare time courses and prognostic values of NPs during early hospitalization (presentation and 24 and 48 hours) with values obtained prior to hospital discharge.

### Statistical analysis

Statistical analysis was performed with the SPSS/PC software package (version 16.0; SPSS, Inc., Chicago, IL, USA). A statistical significance level of 0.05 was considered significant. Discrete variables are expressed as counts (percentage) and continuous variables are expressed as mean ± standard deviation or as median and interquartile range (IQR) unless stated otherwise. Frequency comparisons were made using the *t *test, Kruskal-Wallis test, Mann-Whitney *U *test, and chi-square test as appropriate. Receiver operating characteristic (ROC) curves were drawn to quantify the ability of BNP and NT-proBNP to predict outcome. Comparison between areas under the ROC was performed with MedCalc (version 11.2.1; MedCalc, Mariakerke, Belgium). Cox regression analysis was used to identify predictors of mortality. Multivariate analysis, including all candidate variables with a *P *value of not more than 0.1 in the univariate analysis, was carried out to identify independent predictors of survival. The model included age, cTn levels, estimated glomerular filtration rate (eGFR) by the Cockcroft-Gault formula [29], NYHA functional class, and serial measurements of BNP and NT-proBNP as continuous variables. Comparison of time course of BNP and NT-proBNP levels between survivors and non-survivors and between BNP and NT-proBNP in survivors was assessed with analysis of variance (ANOVA) for repeated measures. Kaplan-Meier survival analysis was performed to assess 1-year mortality stratified by tertiles of BNP and NT-proBNP.

## Results

### Mortality and follow-up

Baseline characteristics of the patients are displayed in Table 1. Median duration of hospitalization was 13 days (IQR 8 to 18). Fourteen patients (8%) died during the index hospitalization, and 18 (11%) died during the first 30 days. After 1-year follow-up, a total of 60 patients (35%) died. During 1-year follow-up, there were 34 (20%) hospitalizations for ADHF.

**Table 1:** Baseline characteristics of 171 patients admitted with acute decompensated heart failure

Clinical characteristic	Overall *n *= 171	One-year non-survivors *n *= 60	One-year survivors *n *= 111	*P *value
Female gender, number (percentage)	68 (40)	27 (45)	41 (37)	0.305
Age in years	80	84	77	< 0.001
	(73-85)	(79-89)	(68-83)	
Body mass index, kg/m^2^	26	24	27	0.001
	(23-30)	(22-28)	(25-31)	
Vital signs				
Systolic blood pressure, mm Hg	139	138.5	139.5	0.270
	(117-156)	(111-151)	(121-157)	
Diastolic blood pressure, mm Hg	84	86	83	0.941
	(70-95)	(68-94)	(71-96)	
Heart rate, beats per minute	88	90	87	0.641
	(77-104)	(77-103)	(76-103)	
Echocardiography				
LVEF, percentage	37	40	36	0.579
	(25-55)	(29-51)	(25-65)	
LVEDD, mm	54	51	56	0.037
	(47-61)	(46-58)	(47-65)	
Blood test results, number (percentage)				
Sodium, mmol/L	139	139	139	0.854
	(136-141)	(137-141)	(136-141)	
Potassium, mmol/L	4.2	4.3	4.2	0.577
	(3.8-4.6)	(3.7-4.6)	(3.8-4.6)	
Creatinine, μmol/L	103	131	97	< 0.001
	(80-142)	(92-180)	(75-125)	
Urea, mmol/L	10	14	9	< 0.001
	(7-14)	(10-18)	(7-12)	
Glomerular filtration rate, mL/minute^a^	48	34	60	< 0.001
	(33-70)	(24-48)	(41-83)	
ASAT, U/L	34	36	33	0.049
	(26-45)	(28-53)	(25-42)	
Cardiac troponin T, μg/L	0.02	0.04	0.01	< 0.001
	(0.01-0.04)	(0.01-0.07)	(0.01-0.02)	
BNP, pg/mL	1,315	1,718	973	< 0.001
	(759-2,349)	(1,088-3,042)	(604-1,725)	
NT-proBNP, pg/mL	6,964	11,624	5,840	0.01
	(3,068-14,791)	(5,722-20,597)	(2,617-11,277)	
Co-morbidity, number (percentage)				
Coronary artery disease	62 (37)	37 (33)	25 (42)	0.946
Hypertension	95 (55)	54 (49)	41 (69)	0.324
Chronic heart failure	74 (43)	32 (53)	42 (38)	0.482
Renal dysfunction	62 (37)	34 (57)	28 (25)	0.002
Diabetes mellitus	48 (30)	26 (24)	22 (37)	0.355
Symptoms, number (percentage)				
Dyspnea				0.180
NYHA II	2 (1)	0	2 (2)	
NYHA III	69 (40)	18 (30)	51 (46)	
NYHA IV	92 (54)	41 (69)	51 (46)	
Chest pain	47 (37)	16 (31)	31 (41)	0.250
Weight gain	56 (44)	22 (42)	34 (45)	0.863
Orthopnea	81 (63)	29 (56)	52 (68)	0.843
Paroxysmal nocturnal dyspnea	59 (46)	16 (31)	43 (57)	0.133
Etiology of heart failure, number (percentage)				
Ischemic heart disease	41 (24)	10 (16)	31 (29)	0.295
Hypertensive heart disease	60 (35)	20 (32)	40 (36)	0.815
Valvular heart disease	40 (23)	22 (37)	18 (16)	0.161
Idiopathic heart disease	22 (13)	4 (6)	18 (16)	0.267
Other^b^	8 (5)	4 (9)	4 (3)	0.823
Electrocardiogram, number (percentage)				
Sinus rhythm	83 (49)	35 (46)	25 (48)	0.822
Atrial fibrillation/flutter	44 (26)	20 (33)	24 (21)	0.423
QRS duration, milliseconds	112 (95-151)	125 (100-154)	110 (92-144)	0.210
Admission medication, number (percentage)				
Aspirin	68 (40)	23 (39)	45 (41)	0.940
Clopidogrel	16 (10)	4 (7)	12 (11)	0.607
Oral anticoagulation	67 (40)	25 (42)	42 (38)	0.492
Beta-blocker	120 (70)	39 (65)	81 (73)	0.103
ACE inhibitor	116 (68)	33 (55)	83 (75)	0.025
Angiotensin II receptor blocker	67 (40)	18 (30)	49 (44)	0.103
Calcium channel-blocker	68 (40)	20 (33)	48 (43)	0.341
Diuretics	145 (85)	52 (87)	93 (84)	0.431
Aldosterone antagonist	20 (12)	6 (10)	14 (13)	0.856
Digoxin	10 (6)	5 (8)	5 (5)	0.274
Nitrates	41 (24)	15(25)	26 (24)	0.836
Heart failure medication 0 to 72 hours				
Furosemide, mg	40 (20-80)	40 (0-60)	40 (20-100)	0.095
Torasemide, mg	30 (10-80)	30 (20-70)	20 (0-130)	0.639
Nitrates^c^, mg/24 hours	40 (15-83)	40 (20-117)	30 (10-60)	0.121
Discharge medication, number (percentage)				
Aspirin	61 (36)	15 (25)	46 (42)	0.033
Clopidogrel	18 (11)	4 (7)	14 (13)	0.228
Oral anticoagulation	73 (43)	20 (33)	53 (48)	0.070
Beta-blocker	116 (68)	28 (47)	88 (80)	< 0.001
ACE inhibitor	111 (65)	32 (54)	79 (71)	0.052
Angiotensin II receptor blocker	49 (29)	9 (15)	40 (36)	0.006
Calcium channel-blocker	30 (18)	9 (15)	21 (19)	0.617
Diuretics	147 (86)	44 (73)	103 (93)	< 0.001
Aldosterone antagonist	27 (16)	9 (15)	18 (16)	0.836
Digoxin	18 11)	6 (10)	12 (11)	0.869
Nitrates	62 (37)	22 (37)	40 (36)	0.745

Values are presented as median (interquartile range) unless stated otherwise. ^a^Using the Cockcroft and Gault formula [29]; ^b^including hypertrophic obstructive cardiomyopathy, myocarditis, and alcoholic cardiomyopathy; ^c^usually applied transdermally. ACE, angiotensin-converting enzyme; ASAT, aspartate aminotransferase; BNP, B-type natriuretic peptide; LVEDD, left ventricular end diastolic diameter; LVEF, left ventricular ejection fraction; NT-proBNP, N-terminal B-type natriuretic peptide; NYHA, New York Heart Association.

### Clinical characteristics and outcome

Patients who died during 1-year follow-up had lower body mass index (BMI) (*P *= 0.001) and eGFR (*P *< 0.001) levels and higher cTn (*P *< 0.001), ASAT (*P *< 0.05), BNP (*P *< 0.001), and NT-proBNP (*P *= 0.01) levels. Treatment with aspirin (*P *= 0.033), beta-blocker (*P *< 0.001), angiotensin II receptor blocker (ARB) (*P *= 0.006), or diuretics (*P *< 0.001) was higher in survivors (Table 1).

### Prognostic value of serial BNP and NT-proBNP measurements

#### One-year all-cause mortality

**Figure 1:**
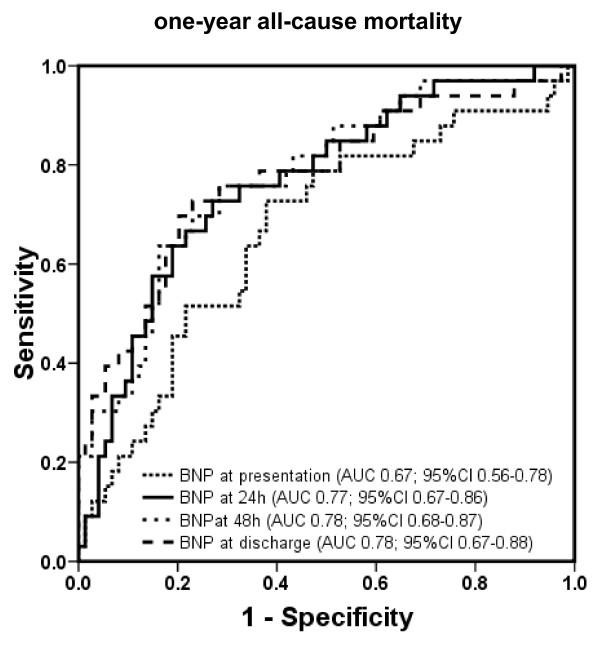
**Receiver operating characteristic curves displaying accuracy of presentation, 24-hour, 48-hour, and discharge B-type natriuretic peptide (BNP) levels to predict 1-year all-cause mortality in patients with acute decompensated heart failure (*n *= 171)**. AUC, area under the curve; CI, confidence interval.

**Figure 2:**
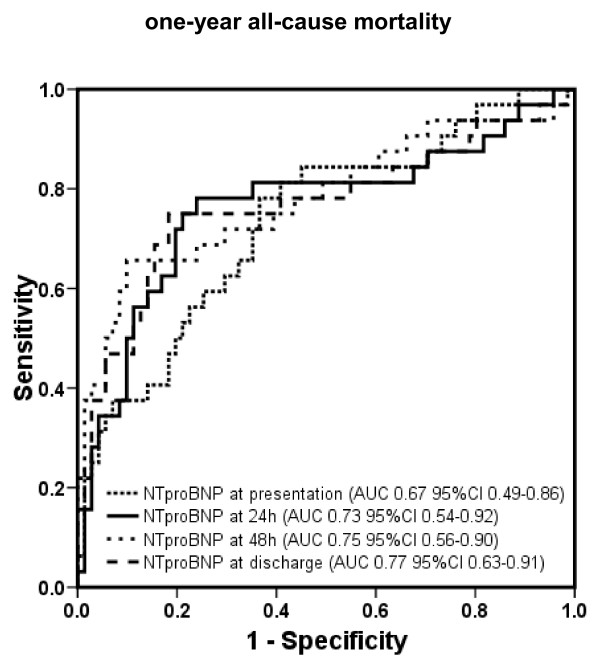
**Receiver operating characteristic curves displaying accuracy of presentation, 24-hour, 48-hour, and discharge N-terminal pro B-type natriuretic peptide (NT-proBNP) levels to predict 30-day all-cause mortality in patients with acute decompensated heart failure (*n *= 171)**. AUC, area under the curve; CI, confidence interval.

The areas under the ROC curve and 95% confidence interval (CI) of BNP for prediction of 1-year mortality at admission, 24 hours, 48 hours, and prior to discharge are displayed in Figure 1 (*P *= not significant [ns] between different time points). Areas under the ROC curve and 95% CI of NT-proBNP at the determined points in time are shown in Figure 2 (*P *= ns between different time points).

#### Thirty-day all-cause mortality

**Figure 3:**
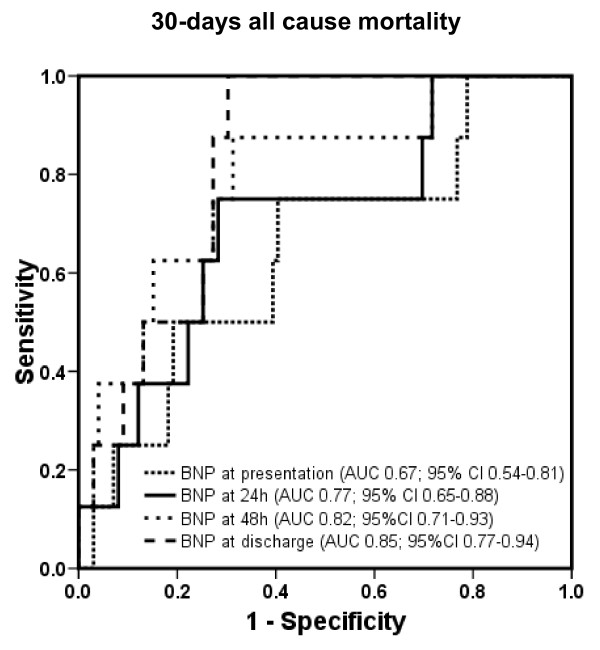
**Receiver operating characteristic curves displaying accuracy of presentation, 24-hour, 48-hour, and discharge B-type natriuretic peptide (BNP) levels to predict 1-year heart failure hospitalization in patients with acute decompensated heart failure (*n *= 171)**. AUC, area under the curve; CI, confidence interval.

**Figure 4:**
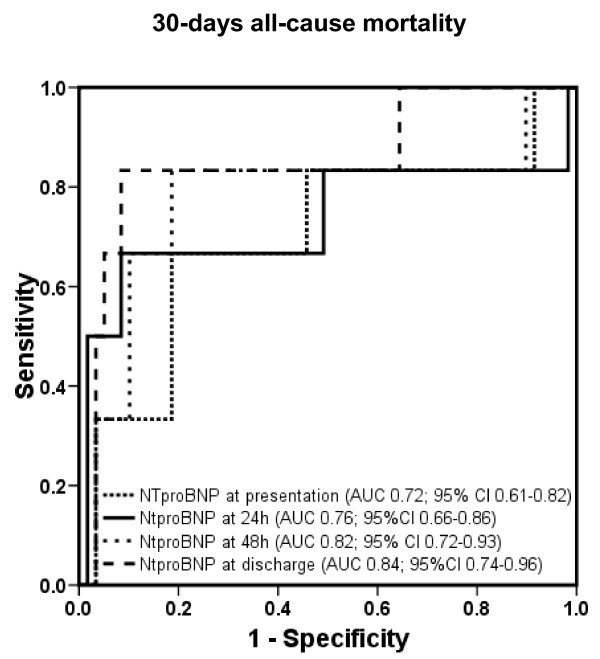
**Receiver operating characteristic curves displaying accuracy of presentation, 24-hour, 48-hour, and discharge N-terminal pro B-type natriuretic peptide (NT-pro BNP) levels to predict 1-year all-cause mortality in patients with acute decompensated heart failure (*n *= 171)**. AUC, area under the curve; CI, confidence interval.

The areas under the ROC curve and 95% CI for BNP for prediction of 30-day mortality at the determined points in time are displayed in Figure 3 (*P *= 0.025 between area under the ROC curve at admission and at discharge, and *P *= ns between all other different time points). The corresponding values of NT-proBNP are shown in Figure 4 (*P *= ns between different time points).

#### One-year heart failure hospitalization

**Figure 5:**
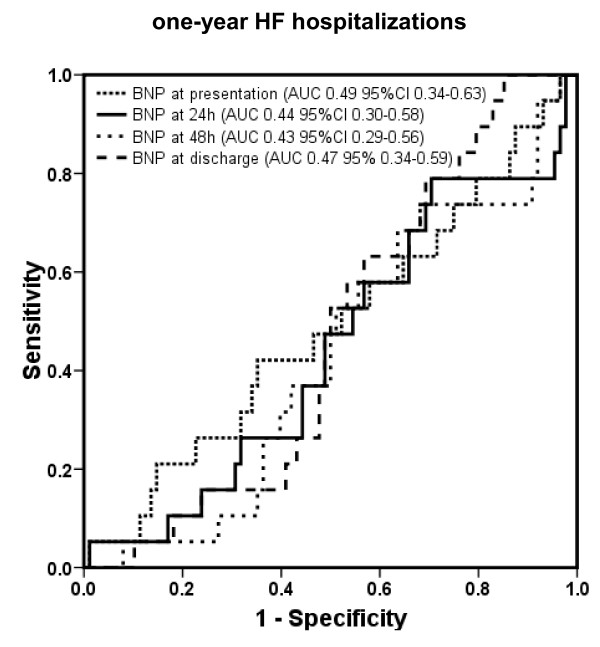
**Receiver operating characteristic curves displaying accuracy of presentation, 24-hour, 48-hour, and discharge B-type natriuretic peptide (BNP) levels to predict 30-day all-cause mortality in patients with acute decompensated heart failure (HF) (*n *= 171)**. AUC, area under the curve; CI, confidence interval.

**Figure 6:**
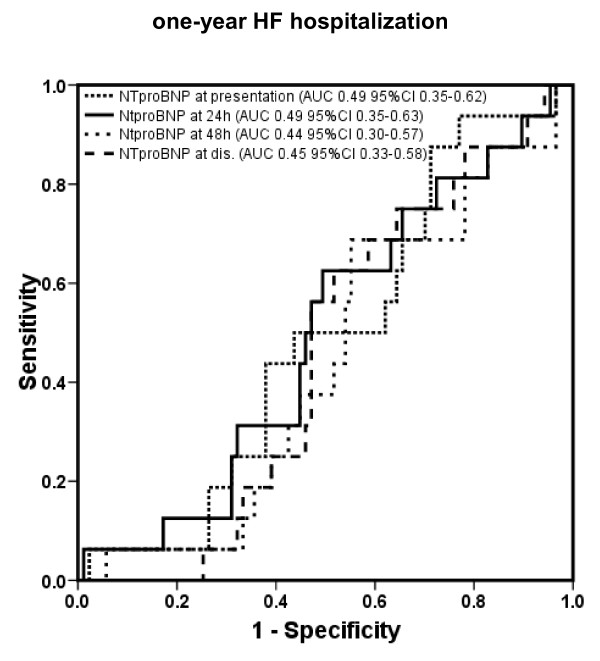
**Receiver operating characteristic curves displaying accuracy of presentation, 24-hour, 48-hour, and discharge N-terminal pro B-type natriuretic peptide (NT-pro BNP) levels to predict 1-year heart failure (HF) hospitalization in patients with acute decompensated HF (*n *= 171)**. AUC, area under the curve; CI, confidence interval.

The areas under the ROC curve and 95% CI of BNP for prediction of 1-year HF hospitalization are shown in Figure 5 (*P *= ns between all different time points), and the corresponding values of NT-proBNP are shown in Figure 6 (*P *= ns between different time points).

### Individual time course of BNP and NT-proBNP in survivors and non-survivors

**Figure 7:**
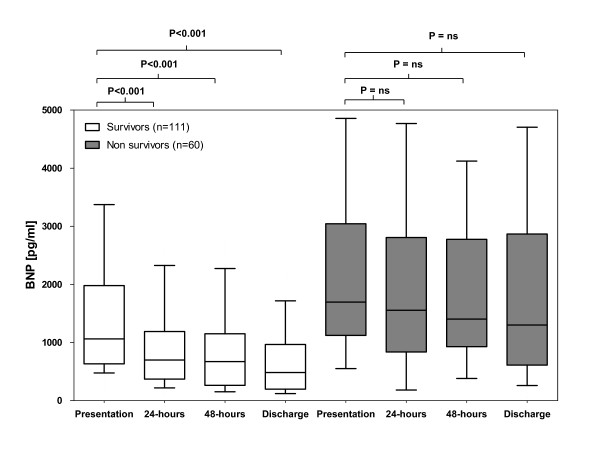
**B-type natriuretic peptide (BNP) levels at admission, at 24 hours, at 48 hours, and at discharge in 1-year survivors and non-survivors with acute decompensated heart failure**. ns, not significant.

BNP levels were higher in 1-year non-survivors compared with 1-year survivors during the entire course of hospitalization (all *P *< 0.001) (Figure 7). In 1-year survivors, BNP levels declined during the course of hospitalization (34% between presentation and 24 hours, *P *< 0.001; 37% between presentation and 48 hours, *P *< 0.001; and 55% between presentation and discharge, *P *< 0.001) (Figure 7). In 1-year non-survivors, BNP levels showed no significant change from admission through the course of hospitalization (Figure 7).

**Figure 8:**
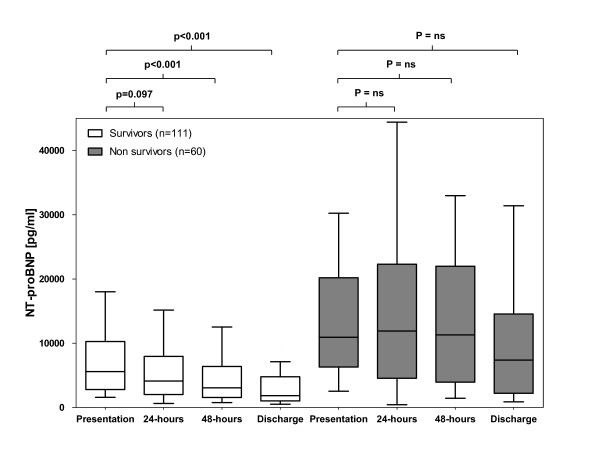
**N-terminal pro B-type natriuretic peptide (NT-proBNP) levels at admission, at 24 hours, at 48 hours, and at discharge in 1-year survivors and non-survivors with acute decompensated heart failure**. ns, not significant.

NT-proBNP levels were higher in 1-year non-survivors compared with survivors during the entire hospitalization (all *P *< 0.001) (Figure 8). In 1-year survivors, NT-proBNP levels declined during the course of hospitalization (27% between presentation and 24 hours, *P *= 0.097; 45% between presentation and 48 hours, *P *< 0.001; and 67% between presentation and discharge, *P *< 0.001) (Figure 8). In 1-year non-survivors, no significant change of NT-proBNP levels compared with baseline occurred during hospitalization (Figure 8).

### Direct comparison between time course of BNP with NT-proBNP at different points in time

Direct comparison of time courses between BNP and NT-proBNP in 1-year survivors by two-way ANOVA for repeated measures demonstrated a difference during the first 24 hours of hospitalization (*P *= 0.003). However, comparison of time courses between BNP and NT-proBNP from presentation to 48 hours (*P *= 0.332) or from presentation to discharge (*P *= 0.114) showed no difference. The optimal cut-point, assessed by maximizing the sum between sensitivity and specificity for BNP and NT-proBNP to discriminate between 1-year survivors and non-survivors at different time points, is displayed in Table 2.

**Table 2:** Optimal BNP and NT-proBNP cut-point assessed by maximizing the sum between sensitivity and specificity to discriminate between 1-year survivors and non-survivors at different time points

Natriuretic peptide (NP)	NP value, pg/mL	Sensitivity, percentage	Specificity, percentage	LR+	LR-
BNP at 24 hours	1,223	65	76	2.7	0.46
BNP at 48 hours	1,027	76	71	2.5	0.34
BNP at discharge	921	72	74	2.9	0.37
NT-proBNP at 24 hours	8,229	69	77	3.1	0.39
NT-proBNP at 48 hours	7,617	72	81	3.9	0.34
NT-proBNP at discharge	7,042	61	90	6.7	0.43

BNP, B-type natriuretic peptide; LR+, positive likelihood ratio; LR-, negative likelihood ratio; NT-proBNP, N-terminal B-type natriuretic peptide.

### Survival analyses

#### One-year mortality

**Figure 9:**
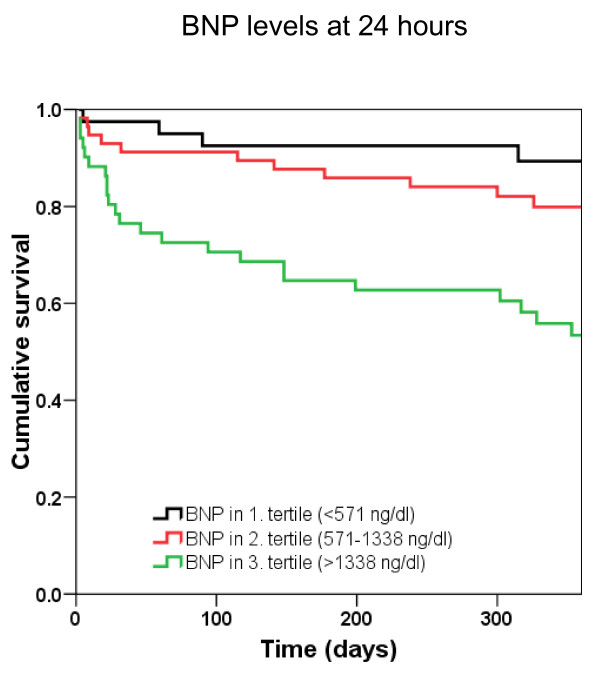
**Kaplan-Meier analysis displaying 1-year mortality stratified by tertiles of B-type natriuretic peptide (BNP) levels at 24 hours**.

**Figure 10:**
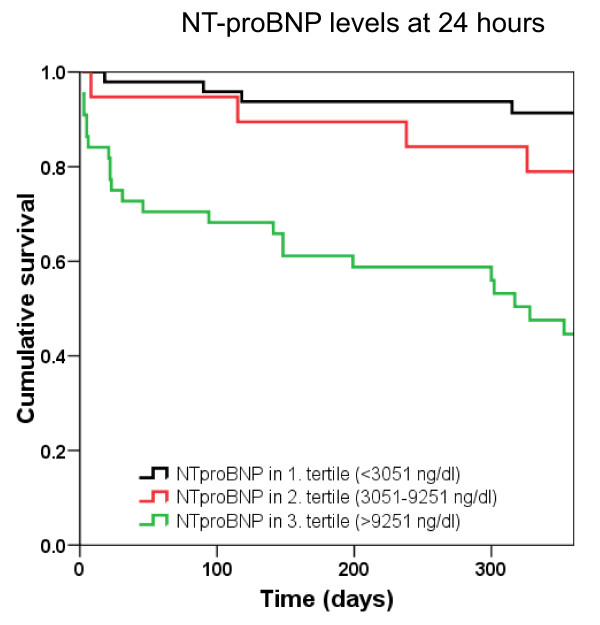
**Kaplan-Meier analysis displaying 1-year mortality stratified by tertiles of N-terminal pro B-type natriuretic peptide (NT-pro BNP) levels at 24 hours**.

Univariate analysis demonstrated that 1-year mortality was predicted by age (per 10-year increase in hazard ratio [HR] 2.49, *P *< 0.001), cTn (HR 18, *P *< 0.001), eGFR (HR 0.96, *P *< 0.001), NYHA functional class (HR 2.1, *P *= 0.009), BNP at 24 hours (per 100 pg/mL increase in HR 1.03, *P *< 0.001), BNP at 48 hours (HR 1.05, *P *< 0.001), BNP at discharge (HR 1.03, *P *< 0.001), NT-proBNP at 24 hours (per 1,000 pg/mL increase in HR 1.04, *P *< 0.001), NT-proBNP at 48 hours (HR 1.06, *P *< 0.001), and NT-proBNP at discharge (HR 1.06, *P *< 0.001). The results of the multivariate analysis models, including age (per 10-year increase), cTn, eGFR, NYHA functional class, and serial BNP (per 100 pg/mL increase) or NT-proBNP (per 1,000 pg/mL increase) levels at different time points are displayed in Table 3. Kaplan-Meier survival analysis was performed to assess 1-year mortality stratified by tertiles of BNP and NT-proBNP determined at 24 hours. Figures 9 and 10 show that both BNP and NT-proBNP in the highest tertile were associated with a higher 1-year mortality compared with levels found in the first or second tertile (*P *< 0.001 by log rank).

**Table 3:** Independent predictors of 1-year mortality by Cox proportional hazards regression in patients admitted with acute decompensated heart failure (*n *= 171)

	Mortality (*n *= 60)	
Baseline variables	HR (95% CI)	*P *value
Multivariable Cox regression model including BNP at 24 hours		
Age, per 10 years increase	1.96 (1.3-3.1)	0.003
Troponin T	15 (3-60)	< 0.001
Glomerular filtration rate	0.99 (0.98-1)	0.375
NYHA functional class	1.52 (0.76-3)	0.236
BNP at 24 hours, per 100 pg/mL increase	1.02 (1.01-1.04)	0.013
Multivariable Cox regression model including NT-proBNP at 24 hours		
Age, per 10 years	1.86 (1.2-3)	0.010
Troponin T	10 (2-53)	0.006
Glomerular filtration rate	0.99 (0.97-1)	0.275
NYHA functional class	1.45 (0.72-2.94)	0.302
NT-proBNP at 24 hours, per 1,000 pg/mL increase	1.01 (0.99-1.04)	0.230
Multivariable Cox regression model including BNP at 48 hours		
Age, per 10 years increase	2 (1.23-3.25)	0.005
Troponin T	6.12 (0.3-13)	0.207
Glomerular filtration rate	0.98 (0.97-1)	0.061
NYHA functional class	1.20 (0.62-2.37)	0.586
BNP at 48 hours, per 100 pg/mL increase	1.03 (1.01-1.06)	0.002
Multivariable Cox regression model including NT-proBNP at 48 hours		
Age, per 10 years	1.74 (1.07-2.87)	0.029
Troponin T	12.70 (0.39-19)	0.869
Glomerular filtration rate	0.98 (0.96-1)	0.075
NYHA functional class	1.35 (0.62-2.95)	0.447
NT-proBNP at 48 hours, per 1,000 pg/mL increase	1.03 (0.99-1.07)	0.063
Multivariable Cox regression model including BNP at discharge		
Age, per 10 years increase	1.89 (1.14-3.14)	0.014
Troponin T	16.90 (0.3-78)	0.148
Glomerular filtration rate	0.98 (0.97-1)	0.061
NYHA functional class	1.20 (0.62-2.36)	0.586
BNP at discharge, per 100 pg/mL increase	1.02 (1.01-1.03)	< 0.001
Multivariable Cox regression model including NT-proBNP at discharge		
Age, per 10 years	3.30 (1.31-8.24)	0.011
Troponin T	15.76 (0.5-61)	0.234
Glomerular filtration rate	1.01 (0.96-1.05)	0.832
NYHA functional class	3.50 (0.74-16)	0.016
NT-proBNP at discharge, per 1,000 pg/mL increase	1.07 (1.01-1.13)	0.016

BNP, B-type natriuretic peptide; CI, confidence interval; HR, hazard ratio; NT-proBNP, N-terminal B-type natriuretic peptide; NYHA, New York Heart Association.

#### Thirty-day mortality

Univariate analysis demonstrated that 30-day mortality was predicted by age (per 10-year increase in HR 1.76, *P *= 0.045), admission systolic blood pressure (HR 0.98, *P *= 0.036), cTn (HR 13.5, *P *< 0.001), eGFR (HR 0.97, *P *= 0.011), BNP at admission (per 100 pg/mL increase in HR 1.02, *P *= 0.043), BNP at 24 hours (HR 1.03, *P *= 0.001), BNP at 48 hours (HR 1.05, *P *< 0.001), BNP at discharge (HR 1.03, *P *< 0.001), NT-proBNP at 24 hours (per 1,000 pg/mL increase in HR 1.04, *P *= 0.017), NT-proBNP at 48 hours (HR 1.06, *P *= 0.001), and NT-proBNP at discharge (HR 1.06, *P *= 0.009). We built multivariate analysis models, including age (per 10-year increase), admission systolic blood pressure, cTn, eGFR, and serial BNP (per 100 pg/mL increase) or NT-proBNP (per 1,000 pg/mL increase) levels at different time points. At 24 hours, 48 hours, and discharge among cTn levels, BNP independently predicted 30-day mortality. NT-proBNP levels at 24 hours and 48 hours could not predict 30-day mortality by multivariate analysis. There was a strong trend for NT-proBNP levels at discharge to independently predict 30-day mortality (*P *= 0.05).

#### One-year heart failure hospitalization

Neither BNP nor NT-proBNP at any measurement time point was able to independently predict 1-year HF hospitalization.

## Discussion

In this study, we determined the prognostic value of serial BNP and NT-proBNP measurements and their accuracy to predict 1-year all-cause mortality, 30-day all-cause mortality, and 1-year HF hospitalization in patients presenting with ADHF. We report five major findings: First, BNP and NT-proBNP levels in 1-year as well as in 30-day non-survivors were higher at presentation and remain higher during the entire course of hospitalization. Second, in 1-year and 30-day survivors, BNP and NT-proBNP levels gradually decreased during the course of hospitalization, whereas in non-survivors, BNP and NT-proBNP levels demonstrated no significant change. Thereby, BNP levels decreased more rapidly than NT-proBNP between presentation and 24 hours. Accordingly, the accuracy of BNP and NT-proBNP to predict 1-year and 30-day mortality increased during the course of hospitalization. Third, at 24 hours, 48 hours, and discharge, BNP levels independently predicted 1-year and 30-day mortality in multivariate analysis whereas only pre-discharge NT-proBNP levels independently predict 1-year mortality. Fourth, neither BNP nor NT-proBNP at any determined point in time could reliably predict 1-year HF hospitalizations. Fifth, the accuracy of BNP to predict 1-year mortality by ROC analysis at 24 hours was comparable to values already obtained at 48 hours or at hospital discharge. This observation suggests that measurement of BNP at 24 hours may be suitable for early assessment of prognosis and consecutive intensification or change of treatment in those patients with continuously elevated levels. These findings are of major clinical importance.

According to other studies, NP levels were higher in patients who died or experienced cardiovascular events. In patients with a favorable outcome, NPs decreased during the course of hospitalization, presumably as a positive response to HF therapy [15,24,28,30]. This decline in NPs was delayed in comparison with improvement of clinical symptoms and hemodynamic parameters and usually was first observed at 24 hours after admission [24-26,28]. In patients with adverse outcome, NP levels remained elevated despite medical therapy, providing valuable prognostic information [15,24,28,30]. Most studies claimed that the best time to predict outcome by measurement of NP was prior to hospital discharge [24,27,28]. Logeart and colleagues [28] examined the prognostic value of serial BNP measurements in patients with ADHF and found elevated pre-discharge BNP levels to be the strongest independent predictor of death or readmission for HF. Comparable results were demonstrated by Cohen-Solal and colleagues [31] in a large trial of ICU patients admitted with ADHF. In the latter study, a BNP decrease of greater than 30% between admission and day 5 independently predicted survival. In our study, we could confirm these results for 1-year survival for a BNP decrease of greater than 30% between admission and discharge (HR 0.42 [0.23 to 0.65], *P *= 0.004) but not for NT-proBNP. Also, an NP decrease of greater than 30% between admission and 24 hours or between admission and 48 hours was not predictive for BNP or for NT-proBNP in our study. O'Brien and colleagues [27] examined the prognostic value of admission and pre-discharge levels of NT-proBNP in patients presenting with ADHF. The main finding of this study was that only pre-discharge NT-proBNP levels independently predicted outcome, and this is consistent with our results.

Recently, Di Somma and colleagues [32] could demonstrate that ADHF patients with a discharge BNP level of less than 300 pg/mL and a percentage decrease during hospitalization of greater than 46% had a better outcome compared with patients with a discharge BNP level of greater than 300 pg/mL or a percentage decrease of less than 46% or both. In this study, ROC curves for percentage decrease of BNP levels at 24 hours after hospitalization reliably predicted adverse events (*P *< 0.001), corroborating the results of our study. The area under the curve (AUC) for percentage decrease at discharge in their study was, however, higher compared with percentage decrease at 24 hours.

The clinical value of outcome measurements performed during a late stage of hospitalization or prior to hospital discharge has limitations. Important decisions regarding patient management and treatment strategy, including consultation by a cardiologist, ICU admission, and non-invasive ventilation, must be taken into account at an early stage of hospitalization. A reliable risk stratification parameter that is available earlier could help to mitigate the dismal outcome of patients with ADHF by treatment intensification. Our data suggest that the 1-year prognostic accuracy of BNP levels measured at 24 hours is comparable to levels obtained prior to discharge, which are widely accepted to be excellent [24,27]. Thus, the most significant change in BNP levels affecting 1-year prognostic value seems to occur during the first 24 hours, reflecting a satisfactory response to HF therapy. Simultaneously, owing to their delayed kinetic, NT-proBNP levels in survivors decline more slowly than BNP levels during the first 24 hours. This finding is supported by other studies [27,30]. Di Somma and colleagues [33] has demonstrated a more rapid decline of BNP compared with NT-proBNP in response to therapy in ADHF patients. Bayés-Genís and colleagues [30] examined the prognostic value of the percentage decrease of NT-proBNP during the course of hospitalization in patients with ADHF. In that study, no significant change in NT-proBNP levels during the first 24 hours was observed, confirming the delayed kinetic of NT-proBNP during early hospitalization. This finding is supported by a study by Metra and colleagues [24], who determined serial measurements of NT-proBNP at 6, 12, 24, and 48 hours and at discharge in consecutive patients with ADHF. The earliest significant decline of NT-proBNP levels was observed at 48 hours, followed by stable NT-proBNP levels during the remaining hospitalization. Di Somma and colleagues [34] found a decrease of NT-proBNP of 18.8% during the first 24 hours in a comparable setting.

Several small studies have compared the diagnostic accuracy of BNP and NT-proBNP [33,35-37]. Unfortunately, it is unknown whether BNP and NT-proBNP differ in their utility to risk-stratify patients with ADHF. In our study, no significant difference between the areas under the ROC at the different measured time points was identified between BNP and NT-proBNP. However, at 24 hours, only BNP levels independently predicted mortality by multivariate analysis, suggesting a more sensitive response in patients with a favorable outcome at this early time point. Whether early risk stratification that is based on persistently elevated BNP levels and that is followed by treatment intensification has the potential to improve patient outcome needs to be confirmed in larger prospective trials.

The mean decreases of BNP levels between presentation and 24 hours in 1-year survivors of our study were 34% for BNP and 27% for NT-proBNP levels. We believe that this rapid change in BNP levels, reflecting an adequate response to HF therapy, is a very important, early risk stratification and therapy guidance tool. A lack of this response, given optimal medical treatment, implies a more complex and therapy-refractory disease associated with an adverse long-term outcome. Accordingly, if this change does not occur, treatment intensification should be the consequence. In patients with a comparable decrease in BNP levels (roughly 30% between admission and 24 hours), we would expect a favorable outcome; however, future prospective studies have to evaluate a distinct cut-point to allow a more precise recommendation.

Interestingly, in our study, neither BNP nor NT-proBNP at any determined time point was able to reliably predict 1-year readmission for HF. Previously published studies presuming this finding - including those of Cheng and colleagues [38], who used BNP, or Bettencourt and colleagues [15], who used NT-proBNP - used combined endpoints consisting of all-cause mortality and readmission for HF.

There were some notable differences beyond NPs between 1-year survivors and non-survivors in our study, including lower BMI and eGFR levels and higher cTn and ASAT levels in non-survivors. More 1-year survivors were treated with beta-blocker and ARB, diuretics, or aspirin.

Obese HF patients have a better outcome compared with patients with low BMI [39,40]. The exact mechanism of this survival benefit linked to higher BMI is yet unknown. Suitable explanations for this paradoxical finding may include an increased neurohumoral and cytokine activation found in patients with advanced HF, leading to higher levels of tumor necrosis factor (TNF) and other inflammatory cytokines [41,42]. TNF and inflammatory cytokines may contribute to myocardial damage and thus to a higher mortality [41,42]. Adipose tissue was demonstrated to produce soluble TNF receptors, which might counteract the harmful property of TNF-α on the myocardium cells [43].

Renal dysfunction is a strong and independent predictor of prognosis in the general population as well as in patients with ADHF [44]. The underlying pathophysiology is multifactorial and most probably associated with decreased renal perfusion, atherosclerosis, inflammation, endothelial dysfunction, neurohormonal activation, and in particular venous congestion [45,46].

cTn levels are known to be elevated in a considerable proportion of patients with ADHF (6% to 10% using standard and 92% using high-sensitivity assays) independently of concomitant acute coronary syndrome [47,48]. The mechanisms underlying cTn release in ADHF remain speculative and include subendocardial ischemia leading to myocyte necrosis, cardiomyocyte damage from inflammatory cytokines or oxidative stress, hibernating myocardium, or apoptosis [49]. cTn has excellent predictive properties in patients with ADHF [48,50,51]; thus, not surprisingly, in our cohort, elevated cTn levels are highly predictive for adverse outcome. At 24 hours, cTn levels and age were even better prognosticators of 1-year mortality compared with BNP.

Liver function test abnormalities are common in patients with HF and independently predict adverse outcome [52-54]. In a *post hoc *analysis of the CHARM (Candesartan in Heart Failure: Assessment of Reduction in Mortality) study, Allen and colleagues [54] demonstrated that elevated total bilirubin was the strongest liver function test predictor of cardiovascular death or HF hospitalizations. In our study, ASAT levels were higher in 1-year non-survivors whereas no difference in total bilirubin or albumin could be observed between 1-year survivors and non-survivors. Since patients with cardiogenic shock were not included in our study, passive hepatic congestion due to increased central venous pressure remains the most suitable explanation for this finding.

There was also a notable difference in 'life-saving' discharge medication between 1-year survivors and non-survivors in our study. One-year survivors received more beta-blocker and ARB compared with non-survivors, whereas treatment with ACE (angiotensin-converting enzyme) inhibitors was comparable. Treatment with beta-blocker and ARB is known to improve outcome in patients with HF; accordingly, our results are consistent with these findings [55,56].

### Limitations

Some limitations of our study need to be mentioned. First, our study may have been too small to reach statistical significance for the comparison between BNP and NT-proBNP for 30-day all-cause mortality at all of the different measurement time points. Second, as we recruited consecutive patients, there may be some interindividual heterogeneity regarding doses of nitrates and diuretics applied as treatments were individualized for each patient. Third, as with all observational studies, we can only hypothesize that patient management could be improved by the clinical use of this monitoring tool.

## Conclusions

BNP and NT-proBNP are potent and nearly equivalent predictors of death in patients admitted with ADHF. The ability of BNP and NT-proBNP to predict 1-year and 30-day mortality increases during the course of hospitalization. In 1-year survivors, BNP declines more rapidly than NT-proBNP during the first 24 hours, suggesting a better suitability for early risk stratification during hospitalization compared with NT-proBNP. Timely prognostic information by serial NP measurements may allow clinicians to intensify treatment in selected patients at a very early stage of hospitalization and thus improve prognosis. BNP and NT-proBNP could not reliably predict HF readmission.

## Key messages

• B-type natriuretic peptide (BNP) and N-terminal pro B-type natriuretic peptide (NT-proBNP) are good and nearly equivalent predictors of death in patients admitted with acute decompensated heart failure.

• Prognostic ability of BNP and NT-proBNP increases during the course of hospitalization.

• BNP declines more rapidly than NT-proBNP during the first 24 hours, suggesting a better suitability for early risk stratification.

• BNP and NT-proBNP could not reliably predict heart failure readmission.

## Abbreviations

ADHF: acute decompensated heart failure; ANOVA: analysis of variance; ARB: angiotensin II receptor blocker; ASAT: aspartate aminotransferase; BMI: body mass index; BNP: B-type natriuretic peptide; CI: confidence interval; cTn: cardiac troponin T; ED: emergency department; eGFR: estimated glomerular filtration rate; HF: heart failure; HR: hazard ratio; ICU: intensive care unit; IQR: interquartile range; LVEF: left ventricular ejection fraction; NP: natriuretic peptide; ns, not significant; NT-proBNP: N-terminal pro B-type natriuretic peptide; NYHA: New York Heart Association; ROC: receiver operating characteristic; TNF: tumor necrosis factor.

## Competing interests

CM has received research support from the Swiss National Science Foundation (PP00B-102853), the Swiss Heart Foundation, the Novartis Foundation, the Krokus Foundation, Abbott (Abbott Park, IL, USA), AstraZeneca (London, UK), Biosite (San Diego, CA, USA), Brahms (Hennigsdorf, Germany), Roche (Basel, Switzerland), Siemens (Munich, Germany), and the Department of Internal Medicine of University Hospital Basel as well as speaker honoraria from Abbott, Biosite, Brahms, Roche, and Siemens. All other authors declare that they have no competing interests.

## Authors' contributions

MN made substantial contributions to the conception and design of the study, acquisition of data, analysis and interpretation of data, and drafting of the manuscript. TB contributed to the acquisition of data, the conception and design of the study, and critical revision of the manuscript. MP, TR, RT, HU, TS, NA, MR, JM, CH, and SS contributed to the acquisition of data and the critical revision of the manuscript. CM made substantial contributions to the conception and design of the study, analysis and interpretation of data, and drafting and critical revision of the manuscript. All authors read and approved the final manuscript.
